# Interaction of spin and vibrations in transport through single-molecule magnets

**DOI:** 10.3762/bjnano.2.75

**Published:** 2011-10-18

**Authors:** Falk May, Maarten R Wegewijs, Walter Hofstetter

**Affiliations:** 1 Institut für Theoretische Physik, Johann Wolfgang Goethe-Universität, 60438 Frankfurt/Main, Germany; 2 Max Planck Institute for Polymer Research, 55128 Mainz, Germany; 3 Institut für Theorie der Statistischen Physik, RWTH Aachen, 52056 Aachen, Germany; 4 Peter Grünberg Institut and JARA - Fundamentals of Information Technology, Forschungszentrum Jülich, 52425 Jülich, Germany

**Keywords:** molecular electronic devices, molecular magnets, nanoelectronic devices, quantum dots

## Abstract

We study electron transport through a single-molecule magnet (SMM) and the interplay of its anisotropic spin with quantized vibrational distortions of the molecule. Based on numerical renormalization group calculations we show that, despite the longitudinal anisotropy barrier and small transverse anisotropy, vibrational fluctuations can induce quantum spin-tunneling (QST) and a QST-Kondo effect. The interplay of spin scattering, QST and molecular vibrations can strongly enhance the Kondo effect and induce an anomalous magnetic field dependence of vibrational Kondo side-bands.

## Introduction

Transport measurements on nanometer-sized magnetic systems address the fundamental problem of how a few magnetic atoms in an anisotropic environment respond to an electron current [1]. Such an environment is provided, for instance, by ligand groups holding such atoms together in a single magnetic molecule contacted in a break junction [2–3]. A very similar situation arises for transport through magnetic atoms embedded in a molecular network on an insulating surface in an STM setup [4–5]. Such systems, which for simplicity we shall refer to as single-molecule magnets (SMM), constitute a single, large spin-moment with spin-anisotropy. The interplay with quantum transport provides new possibilities to study and control their molecular magnetism. For instance, tunneling allows access to several charge states of the SMM, which can exhibit enhanced magnetic properties [2]. When such charge states are only virtually accessible, effective spin–spin exchange interaction arises [4–5] and inelastic excitation of the spin moment is possible [2], allowing for time-dependent control [6]. A key result is that in either regime the transport depends sensitively on the magnetic anisotropy of the SMM, which is characterized by spin-quadrupole terms in the Hamiltonian. A further new aspect is the recently demonstrated mechanical tuning of these anisotropy terms in a transport setup [3]. Although the effect of such molecular distortions on magnetization measurements of SMM crystal samples has been addressed [7–9], so far their dynamic effect on transport through an SMM have not been studied. One candidate that may enable the sensitive probing of such a coupling of the SMM *spin* to vibrations is a specific type of Kondo effect induced by quantum spin-tunneling (QST). This QST, through the energy barrier arising from a dominant uni-axial magnetic anisotropy term, relies on the presence of weak transverse anisotropy. Combined with the exchange tunneling to attached electrodes, a QST-Kondo resonance specific to SMMs can arise [10]. One might expect such QST-assisted Kondo transport to be simply suppressed by coupling of the spin to molecular vibrations, as this tends to increase the anisotropy barrier [9]. However, the dynamic effect of vibrational fluctuations and the possible competition between longitudinal and transverse spin–vibration coupling have not been studied so far, even though coupling to vibrations in the Kondo regime has been considered for spin-isotropic molecules [11–14].

In this paper we consider the modulation of the magnetic anisotropy of an SMM by a quantized vibrational mode distorting an SMM with half-integer spin. Strikingly, even *without* static transverse anisotropy, a QST-induced Kondo peak can arise in the differential conductance. This Kondo effect is dynamically generated by vibrational fluctuations which distort the SMM, and thereby allow the spin to fluctuate. More generally, a higher QST-Kondo temperature may result from spin-vibration coupling, which is relevant for experimental investigation of low temperature transport through SMMs. This enhancement of the interplay of Kondo spin scattering and QST by discrete vibrations indicates a possible avenue along which transport and quantum magnetism may be combined with nanomechanical effects.

## Model and method

We consider an SMM strongly coupled to electronic leads at low temperature in the Coulomb blockade regime, where the charge on the SMM only changes virtually. We assume that the spin couples to a local vibrational mode with frequency Ω. The total Hamiltonian reads *H* = *H*_SMM_ + *H*_K_ with

[1]
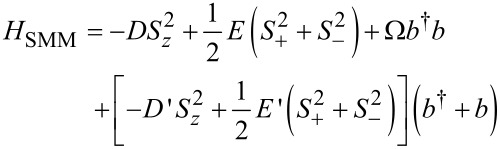


[2]
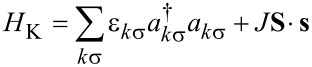


**Figure 1:**
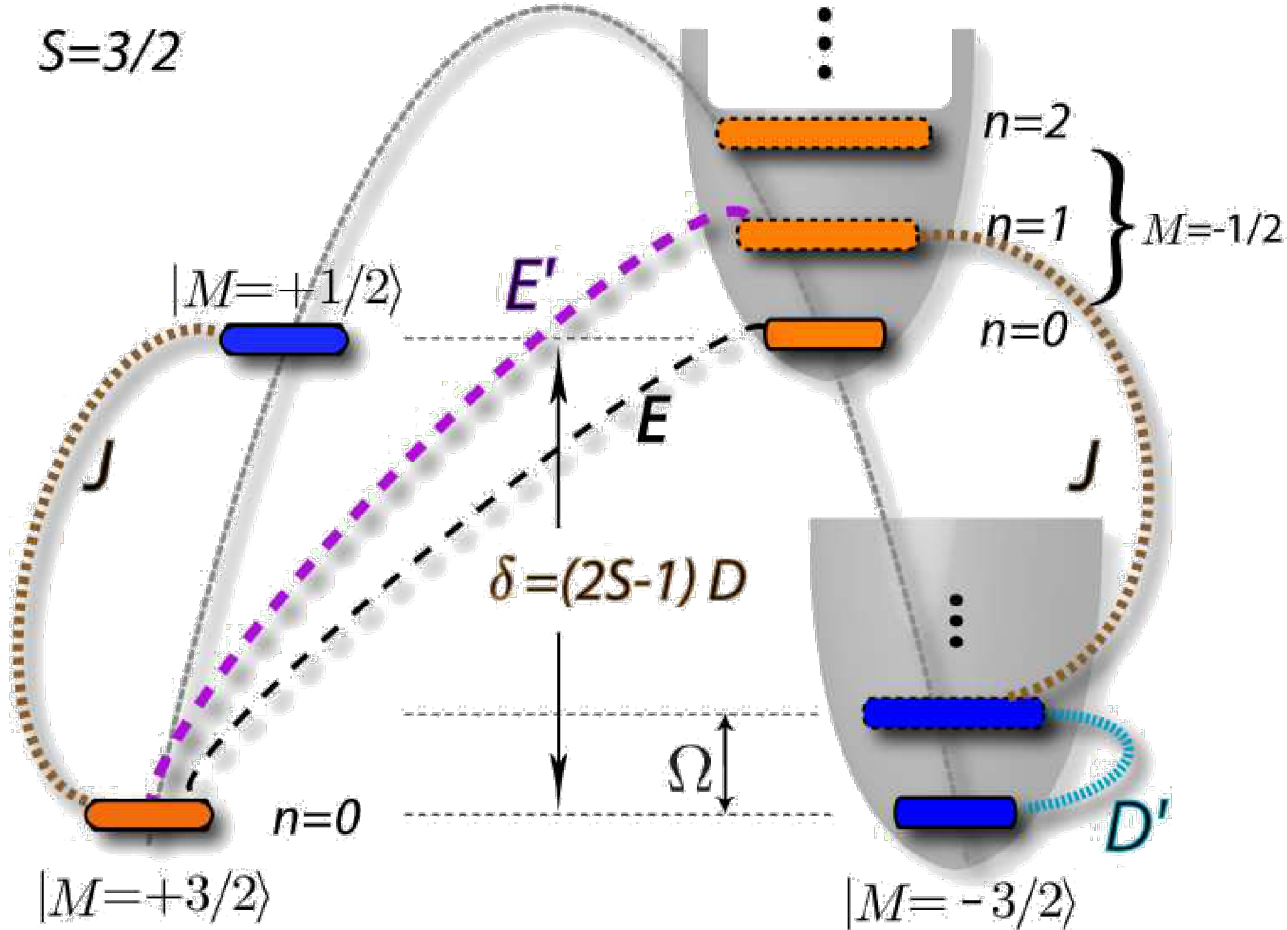
Magneto-mechanical excitation spectrum of the SMM with *S* = 3/2. Magnetic states, lying on an inverted parabola due to the static longitudinal anisotropy (*D*), each have vibrational excitations (*n*) on the upright parabolas. Magnetic transitions induced by Kondo spin-scattering (*J*), static anisotropy (*E*) and longitudinal (*D*′) and transverse (*E*′) spin-vibration coupling are indicated. Magnetic states in the two different Kramers' subspaces are marked blue and orange.

Here *S_z_* is the projection of the molecule’s spin along its easy axis, which we choose to be the *z*-axis and *S*_±_ = *S_x_* ± *iS_y_*. We consider here only half-integer values of the spin magnitude *S*, for which there is a Kondo effect at zero magnetic field [10], and later comment on the integer-spin case. Starting from an isolated molecule, the longitudinal anisotropy *D* splits the eigenstates of *S_z_* into the inverted parabolic magnetic spectrum that is sketched in Figure 1 for the representative case of *S* = 3/2 used throughout this work. The zero-field splitting (ZFS) corresponds to the energy difference between the ground-state and the first magnetic excitation, and equals δ = (2*S* − 1)*D* for *E* = 0 (and *E*′ = *D*′ = 0). The transverse anisotropy *E* breaks the continuous rotational symmetry about the easy-axis of the SMM, thereby causing spin-tunneling through the barrier. As shown in [9], vibrational modes modulate the magnetic anisotropy and can significantly contribute to the observed magnetic splittings. Here we additionally consider the dynamic effects of such coupling by allowing the dominant anisotropy parameters to depend linearly on the vibrational mode coordinate 
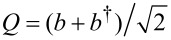
, through the coupling coefficients *D*′ and *E*′. Here the operator *b* (*b*^†^) relaxes (excites) the vibration by one quantum. Thus, when the SMM vibrates, it lowers its symmetry and QST is enhanced. Importantly, this also holds for virtual quantum vibrations. We note that recently such a linear dependence of the *D* parameter on the pitch angle coordinate [15] in the tetra-iron(III) “propeller”-SMM used in [2] was measured. The conduction electron states, represented by the operators 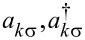 in Equation 2, correspond to even combinations of left and right physical electronic states, and their bandwidth is given by 2*W*. Finally, deep in the Coulomb blockade regime, the interaction of the SMM with the electrodes is given by an isotropic Heisenberg spin-exchange with the conduction band electron spin 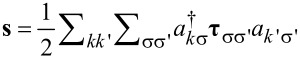 where **τ** is the vector of Pauli matrices. The coupling *J* is assumed to be anti-ferromagnetic, which, as pointed out in [16], depends on the spins of the virtual charge states of the SMM [17–18]. One might expect that coupling to a molecular vibration suppresses the interaction of the SMM with the electrodes due to Franck–Condon overlap, effectively reducing *J*. However, for an isotropic spin of 1/2 it was shown that the effective spin-exchange amplitudes for processes that do not change the vibrational quantum number are hardly modified (in fact they may even be enhanced) and that the amplitude for processes that do change it are suppressed deep in the Coulomb blockade regime [11]. Therefore, we assume *J* to be independent of the vibrational coordinate in Equation 2.

The numerical results presented below for the zero-temperature differential conductance [19],


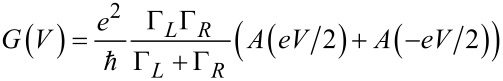


as a function of the bias voltage *V*, were obtained from the SMM spectral function *A*(ω). Here Γ*_L,R_* is the tunnel coupling of the SMM to the left and right electrode. We calculated 
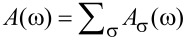
 using the numerical renormalization group (NRG) from the equilibrium spectral function within the T-matrix approach [20]. In linear response to *V, A*(0) provides a numerically exact result for the linear conductance through *G*(0). Moreover, for strong asymmetric coupling of the SMM to the electrodes, the nonequilibrium corrections to the spectral function *A*(ω) are suppressed. In this case, the molecule will predominantly equilibrate with one electrode. Thus, the tunneling results only in a coupling of the spin to the combination of electrode operators that is symmetric with respect to the left/right electrode (our operators *a_k_*_σ_ in Equation 2). Due to the finite bias there is an additional coupling of the even to the odd combination, which is proportional to the voltage *V* and the tunneling amplitude asymmetry. For sufficiently large asymmetry this coupling can be neglected and the function *G*(*V*) provides a good first approximation to the nonlinear conductance as well for the low-lying excitations. From hereon we assume such a large asymmetry in our calculations, which results furthermore in an overall suppression of the conductance without altering the signatures of interest. Therefore the numerical results are normalized to the maximal achievable conductance. For all NRG calculations we used the NRG discretization parameter Λ = 2, kept in total *N*_s_ = 4000 states [21] and included 11 vibrational states on the SMM, which was sufficient to obtain results independent of the vibration number cutoff.

## Static anisotropy

Even without the vibration (*E*′ = *D*′ = 0) or transport (*J* = 0) the SMM eigenstates are not spin eigenstates when *E* ≠ 0. However, for the typical case of moderate transverse anisotropy, *E* < *D*, it still is convenient to label these mixed states by the dominant spin-eigenstate (*M*) in the superposition. Due to the two-fold spin-rotational symmetry of Equation 1 the mixing caused by *E* is only possible within the two subspaces spanned by 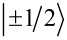,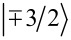 (marked blue and orange in Figure 1). It was shown [10] that upon including exchange spin scattering with conduction band electrons (*J*) the interplay with the QST (generated by *E*) gives rise to a Kondo peak in the differential conductance. Due to the presence of the electrodes, spin fluctuations thus become significant at low temperature despite the presence of the anisotropy barrier of size *DS*^2^ opposing SMM spin reversal. A hallmark of this QST-Kondo effect is that it is suppressed with decreasing ratio of *E*/*D* or increasing *S* (because the barrier grows). This QST-Kondo effect is clearly distinct from the under-screened high-spin Kondo effect [22], which arises for *S* ≥ 1 in the limit without magnetic anisotropy and vibrations (*D* = *E* = *D*′ = *E*′ = Ω = 0). Starting from this limit, introduction of the anisotropy barrier, *D* > 0, splits as well as suppresses the high-spin Kondo peak. The remnants of the high-spin Kondo peak are located at finite bias close to the ZFS scale δ = (2*S* − 1)*D*, with possible renormalization to smaller values for large exchange interaction *J* [2].

These ZFS high-spin Kondo side-peaks have recently been studied in detail in several experiments [2–5]. The QST-Kondo peak, on the other hand, is located at zero bias in the absence of magnetic field. It has, to our knowledge, not been observed experimentally so far. One possible reason for this is that in SMMs typically *E*/*D* < 1 and the QST-Kondo temperature *T*_K_ is suppressed too much, reducing both the height and width of the peak (*without* splitting it).

## Dynamic anisotropy

The anisotropic couplings of the large spin to the vibrational mode *D*′ and *E*′ are, however, also of importance [9], especially if the vibrational mode frequency energy Ω is low. The simplest effect of the longitudinal vibrational coupling *D*′ is a polaronic shift that is different for each magnetic level. For *E* = *E*′ = 0 one can shift the vibrational coordinate *Q* (or the operator *b*) in *H*_SMM_ by an 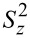-dependent amount, resulting in an effective Hamiltonian with eigenvalues *E_M,n_* = −*DM*^2^ − (*D*′^2^/Ω)*M*^4^ + Ω*n*, where *M* = −*S*,..,*S* and *n* = 0,1,.. are the quantum numbers of the spin and the polaron, respectively. Each SMM eigenstate is thus dressed by vibrational excitations, as sketched in Figure 1. The effective static energy barrier opposing spin inversion is changed in shape and increased in height. This always results in an effectively *enhanced* ZFS δ → Δ,

[3]
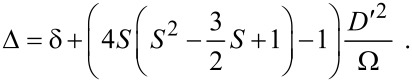


**Figure 2:**
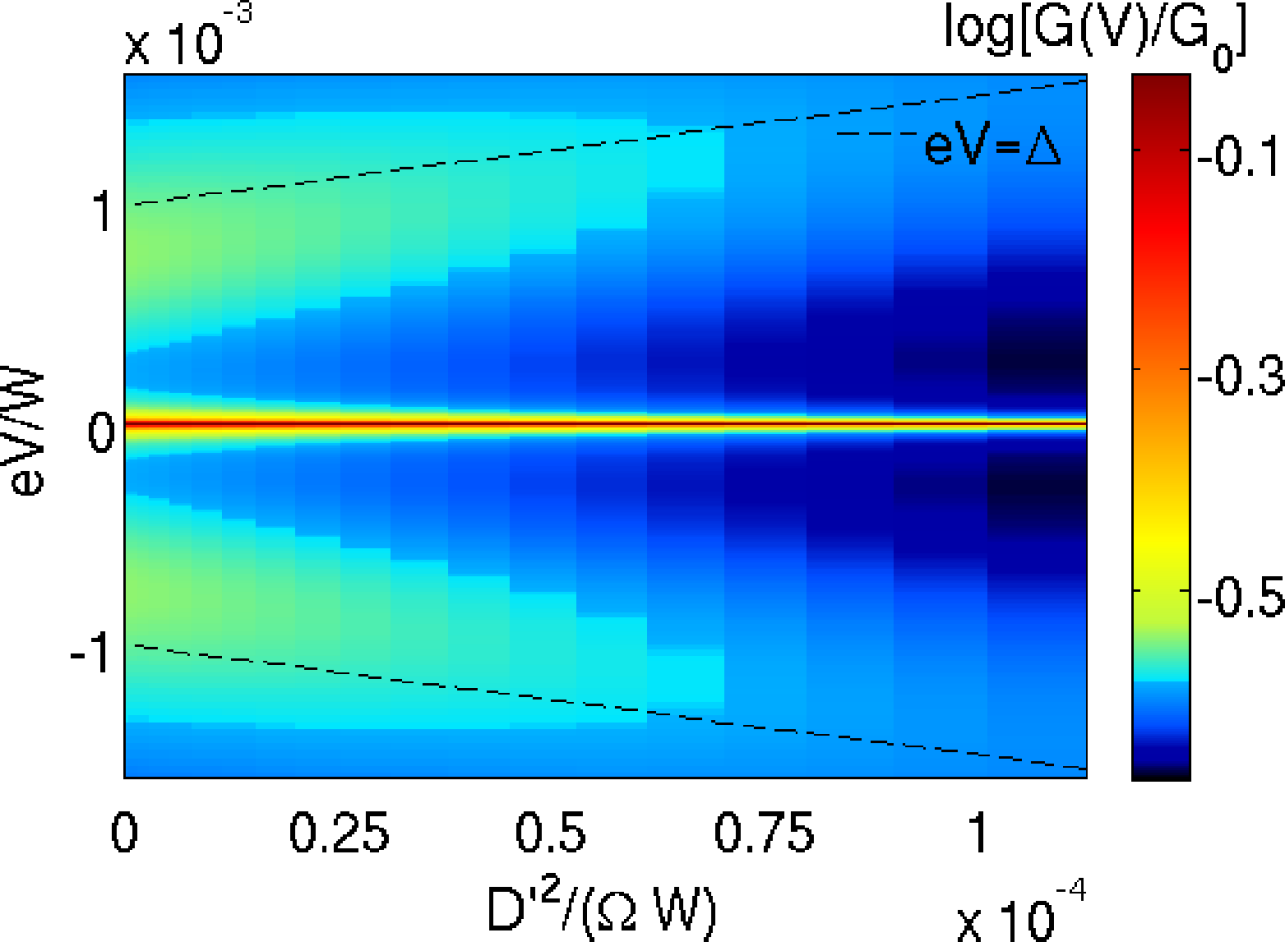
Logarithmic color plot of the SMM differential conductance *G*(*V*) normalized to the value 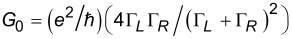 and for *T* = 0. Parameters are *S* = 3/2, *D* = 5 · 10^−4^*W*, *E* = 0.1*D*, *E*′ = 0, Ω = 0.5*D*, *J* = 0.2*W* and *D*′ is varied. The dashed line represents the renormalized ZFS from Equation 3.

Based on this simple picture one may expect that the QST-Kondo effect is suppressed by coupling to vibrations. For fixed *E*′ = 0 this is indeed the case, as can be seen in Figure 2. All reported numerical calculations were performed for parameters whose orders of magnitude relative to the typical bandwidth of several eV were chosen in agreement with known molecular parameters [15] and transport parameters [2]. The only exception to this is for the vibrational parameters *E*′, *D*′ and Ω, for which we performed a systematic study. We focus on low frequency modes on the scale of the anisotropy parameters, taking Ω = 0.5*D*, for which the dynamic effects are most pronounced. We note that in STM setups [4–6] the anisotropy can be on the meV scale. For the spin value we take the next-to-lowest half-integer value *S* = 3/2 , representative of the essential physics. Due to the moderate but nonvanishing static *E* = 0.1*D*, a zero-bias QST-Kondo peak (red) occurs, which is increasingly suppressed with the coupling *D*′ due to an increased barrier. In addition two side-peaks are found at the effective ZFS ω ≈ ±Δ given by Equation 3, as indicated by the dashed lines (and renormalized to a slightly smaller value due to the strong *J*). These peaks are the remnants of the *S* = 3/2 high-spin Kondo effect.

**Figure 3:**
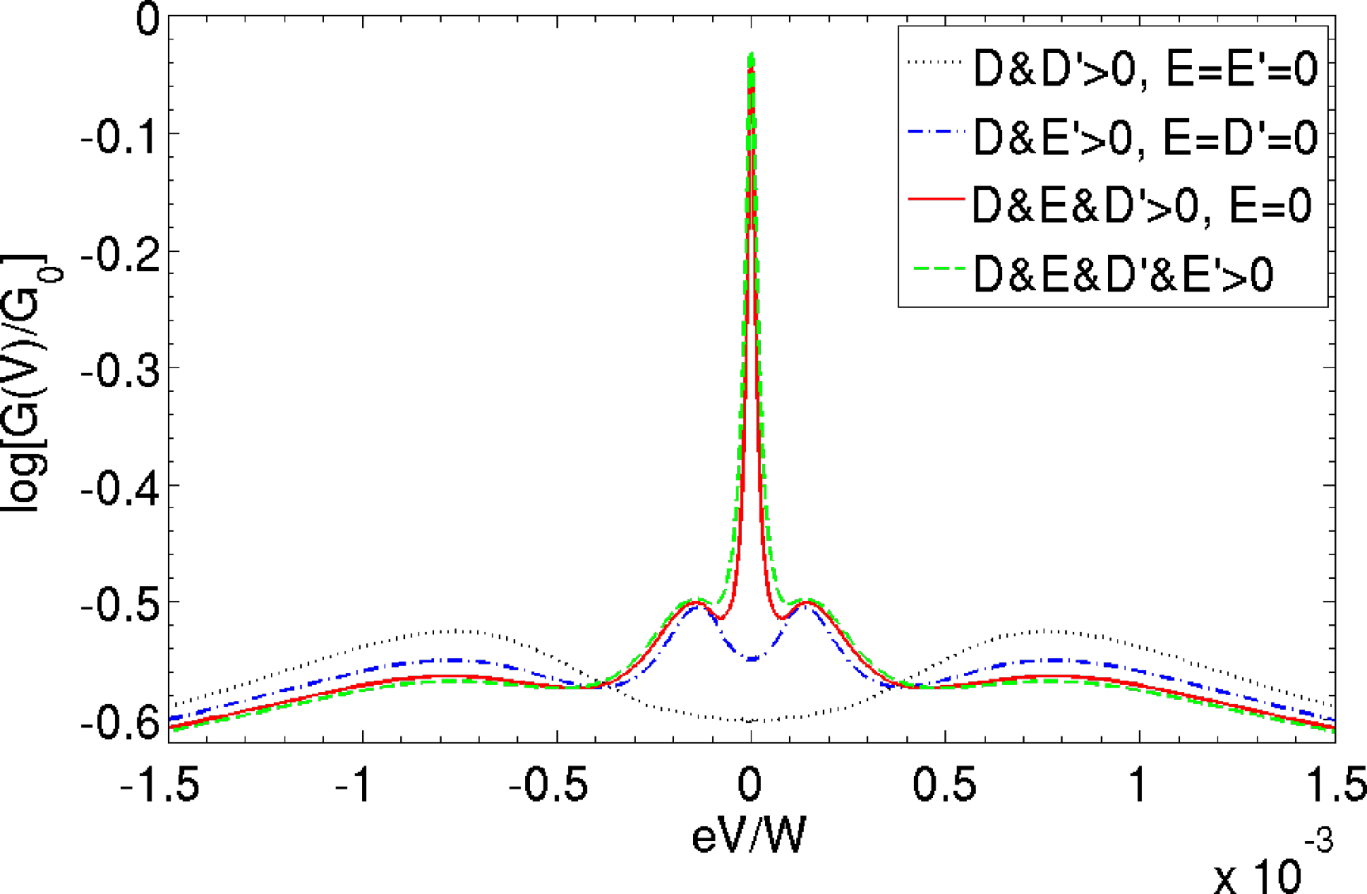
Effect of the spin–vibration coupling on the QST-Kondo peak: SMM differential conductance shown for various combinations of zero and nonzero values of the parameters. Nonzero values used: *D*′ = 0.04*D*, *E*′ = 0.16*D* and *E* = 0.02*D*. Remaining parameters as in Figure 2.

In Figure 3 we now focus on the dynamic effect of the spin–vibration coupling by first considering zero transverse anisotropy (*E* = 0). Without coupling to the vibrations there is no QST-Kondo peak. Switching on spin–vibration coupling of only one type, either *D*′ ≠ 0 and *E*′ = 0 (black), or, *E*′ ≠ 0 and *D*′ = 0 (blue), does not change this result. Only in the latter case, vibrational side-peaks appear in the spectrum at ω = ±Ω (renormalized to smaller value due to strong *J*). Strikingly, when both types of couplings are nonzero, a pronounced QST-Kondo peak appears, even though there is no transverse magnetic anisotropy *E* = 0 (red). This *vibration-induced* QST-Kondo effect is the central result of this work. We now first explain why it requires the presence of both longitudinal and transverse couplings, referring to the processes sketched in Figure 1. The Kondo effect is related to fluctuations between degenerate states of the SMM that are in opposite Kramers’ subspaces [10]. Since *E* = 0, to reach states on the other side of the anisotropy barrier, a vibration-induced spin-tunneling of type *E*′ is required, which however, involves a virtual vibrational excitation. An exchange scattering process (*J*), which changes the Kramers’ subspace, cannot change the vibrational number at low energy [11]. This is why the two processes *E*′ and *J* result only in a QST-Kondo side-peak in Figure 3, which is split at Ω and suppressed due to the inability to reach the vibrational ground-state. Only when a longitudinal spin-coupling *D*′ is present as well, can the virtual vibrational excitation *coherently* reach the ground state and a full *zero-bias* QST-Kondo anomaly can develop, as the red curve in Figure 3 shows. One may say that due to the quantum fluctuations of the vibrational mode of the SMM, the magnetic symmetry is broken in virtual intermediate states, allowing for Kondo exchange scattering [12]. Therefore even for SMMs that have vanishing static *E* due to symmetry, spin-fluctuations may result in pronounced transport features due to the interplay of exchange scattering and intramolecular spin–vibration coupling. Clearly, the vibrational fluctuations can further assist the QST-Kondo effect when it is already present due to static *E*; this results in a higher Kondo temperature as shown in the green curve of Figure 3.

**Figure 4:**
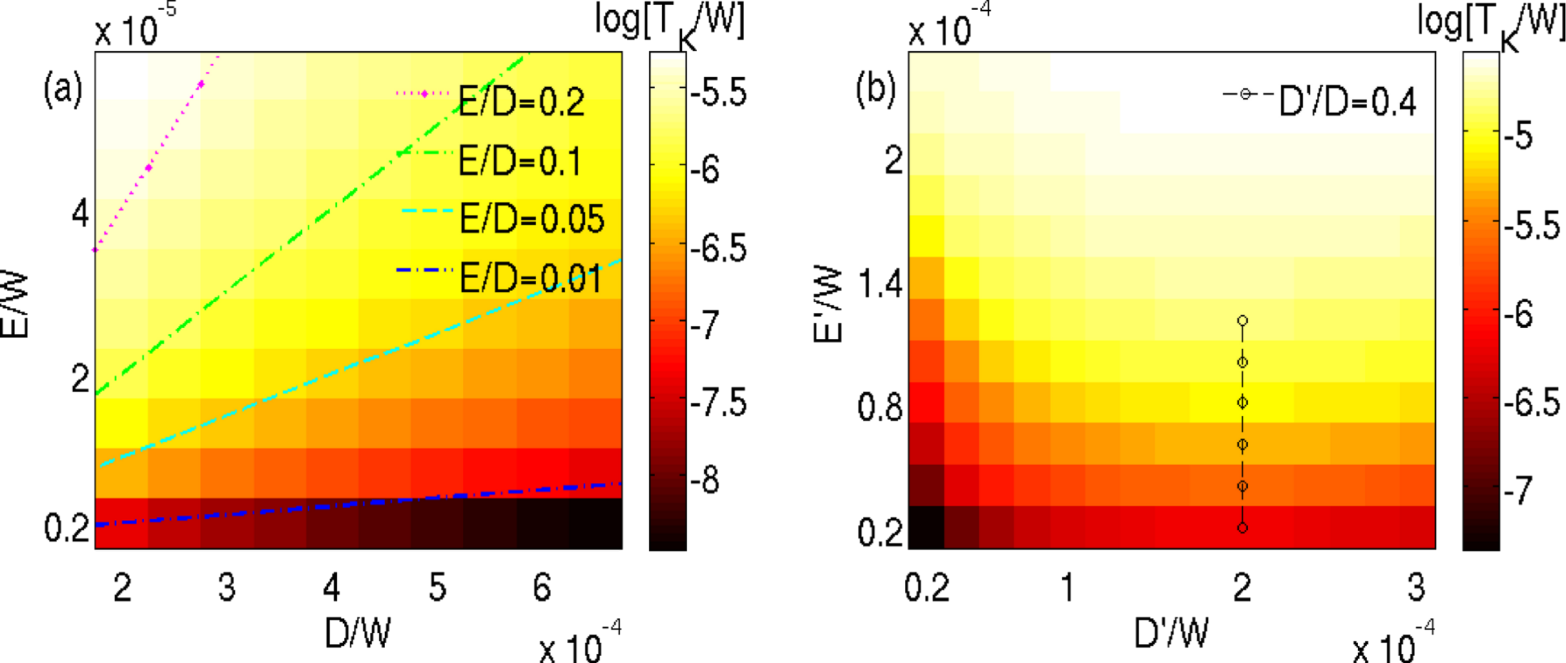
QST-Kondo temperature *T*_K_ in units of bandwidth 2*W* (log color scale) determined from the NRG level flow for *S* = 3/2 and *J* = 0.2*W*. (a) Static QST-Kondo effect: *T*_K_ as a function of the static anisotropy parameters *D, E* without spin-vibration coupling. Dashed contour lines indicate that *T*_K_ increases with the ratio *E*/*D* [10]. (b) Vibration-induced QST-Kondo effect: *T*_K_ as function of spin–vibration couplings *D*′ and *E*′ with Ω = 0.5*D* for static anisotropy, *D* = 5 · 10^−4^*W* and *E* = 0. The dashed line marks the regime *D*′ > 0.4*D* where *D*′ starts to suppress *T*_K_, note the constant *D* here and also the offset: *T_K_* = 0 for either *E*′ = 0 or *D*′ = 0. Results for small finite *E* are similar.

In Figure 4 we show the dependence of the QST-Kondo temperature *T*_K_ on the longitudinal and transverse static anisotropies and their respective vibration couplings *D*′ and *E*′. *T*_K_ grows as both the transverse anisotropies are increased, since they both enhance QST. Whereas *D* always suppresses the QST-Kondo effect (Figure 4a) its fluctuations first enhance *T*_K_ by allowing QST between the Kramers degenerate ground-states. Eventually they will suppress *T*_K_ if the vibrational contribution to the anisotropy barrier *S*^4^*D*′^2^/Ω, protecting the SMM against these processes, increases too much.

**Figure 5:**
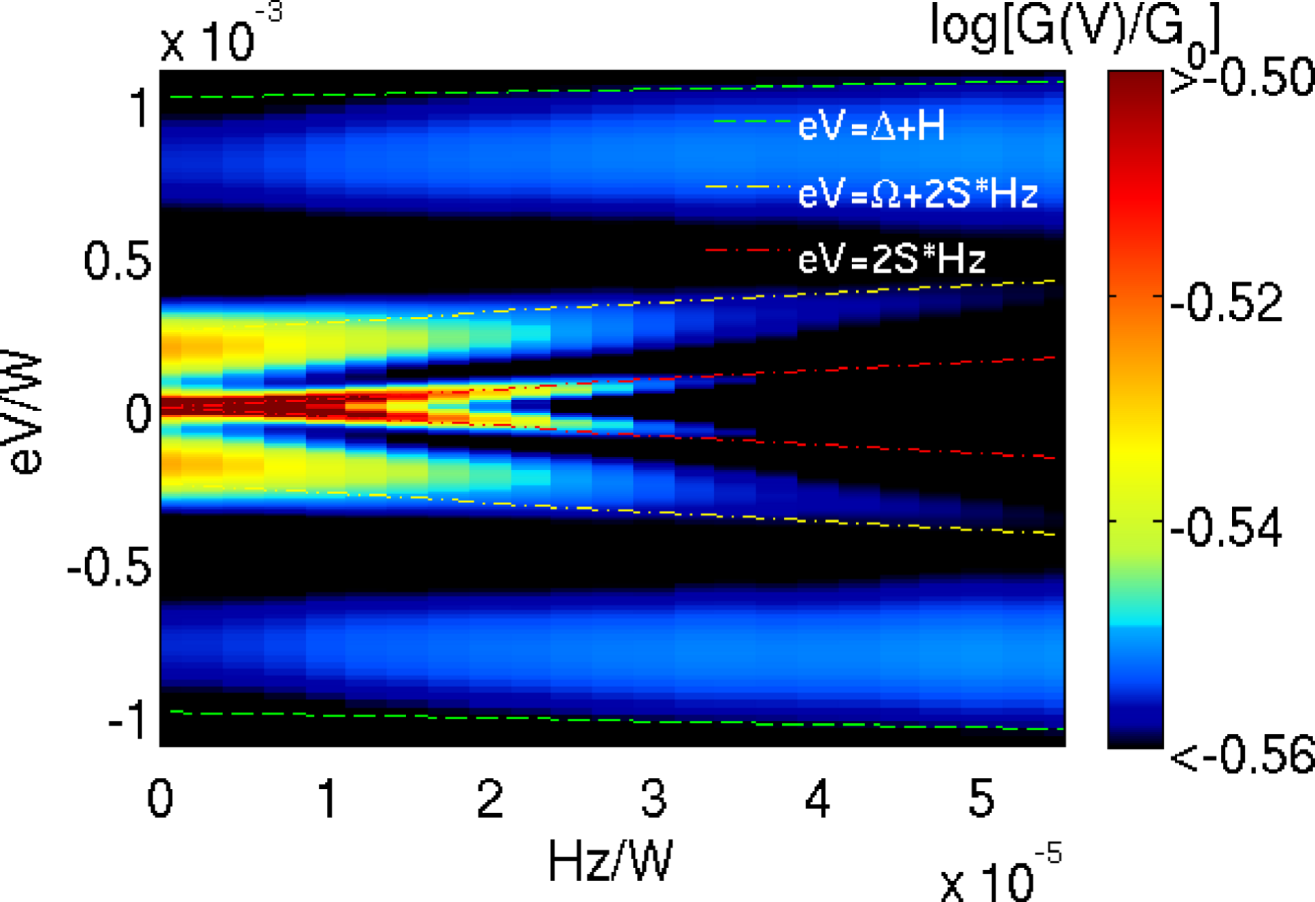
Magnetic field evolution of the differential conductance normalized to *G*_0_ (log color scale) for the parameters of the red curve of Figure 3: *S* = 3/2, *E* = 0.02*D*, *D*′ = 0.04*D* and *E*′ = 0.16*D*. Dashed lines mark the QST-Kondo (red) and vibrational side-peak (yellow) both evolving with an anomalous *g*-factor and zero-field, split, high-spin Kondo peak (blue).

Finally, we show in Figure 5 how the experimentally accessible evolution of the conductance with a magnetic field reveals the different origin of the various peaks. For simplicity, we consider the field to be along the easy axis, as in some experiments [2–3], and we add the Zeeman term −*S_z_H_z_* to *H*_SMM_ in Equation 1, absorbing the *g*-factor into the magnetic field. As the magnetic field is increased, the QST-Kondo peak is weakened and splits with the anomalous *g*-factor, |ω_Kondo_| = 2*SH_z_* [23]. This clearly indicates the origin of the QST-Kondo effect, since the ground-state Kramers’ doublet *M* = ±*S* is split by Δ*M* = ±2*S*. Strikingly, the vibrational side-peaks have the same strong field dependence, as they correspond to a similar transition offset in energy by Ω, that is, |ω_vib_| = Ω + 2*SH_z_*. In contrast to this, the high-spin Kondo peak (ZFS) evolves much slower in the magnetic field, independent of the spin magnitude *S*, thus |ω_ZFS_| =Δ + *H_z_*, signaling that it corresponds only to a transition with Δ*M* = ±1. Comparing the above formulas for the peak evolution, indicated by dashed lines in Figure 5, we conclude that the QST-Kondo and high-spin Kondo effects are distinguishable, especially for SMM with large spin.

Finally, we remark that for an SMM with integer spin *S*, there is no QST-Kondo effect at zero field, but instead a zero-bias conductance dip (for 0 < *E* < *D*). As pointed out in [24] a transverse magnetic field results in a QST-Kondo effect, where similar spin-vibration effects as studied here could occur.

## Conclusion

We have studied the interplay of spin and vibration on the conductance through a single-molecule magnet. Whereas longitudinal coupling to the vibration increases the zero-field splitting and suppresses the quantum spin-tunneling Kondo peak, a vibrationally induced quantum spin-tunneling Kondo effect can occur at zero bias if transverse coupling is present as well. The transition to virtual vibrational excited states and the transverse spin-mixing in these virtual states results in a Kondo effect, even in the absence of static transverse anisotropy. The interplay with vibrations can thus increase the quantum spin-tunneling Kondo temperature for a given static anisotropy, which may motivate further experimental investigation of low temperature transport though single-molecule magnets. The measurable magnetic field evolution of the conductance reveals that vibrational side-bands acquire an anomalous *g*-factor.
